# Suitability and Allocation of Protein-Containing Foods According to Protein Tolerance in PKU: A 2022 UK National Consensus

**DOI:** 10.3390/nu14234987

**Published:** 2022-11-24

**Authors:** Maria Inês Gama, Sarah Adam, Sandra Adams, Heather Allen, Catherine Ashmore, Sarah Bailey, Barbara Cochrane, Clare Dale, Anne Daly, Giana De Sousa, Sarah Donald, Carolyn Dunlop, Charlotte Ellerton, Sharon Evans, Sarah Firman, Suzanne Ford, Francine Freedman, Moira French, Lisa Gaff, Joanna Gribben, Anne Grimsley, Ide Herlihy, Melanie Hill, Farzana Khan, Nicola McStravick, Chloe Millington, Nicola Moran, Camille Newby, Patty Nguyen, Janet Purves, Alex Pinto, Júlio César Rocha, Rachel Skeath, Amy Skelton, Simon Tapley, Alison Woodall, Carla Young, Anita MacDonald

**Affiliations:** 1 Birmingham Women’s and Children’s NHS Foundation Trust, Birmingham Children’s Hospital, Steelhouse Lane, Birmingham B4 6NH, UK; 2 Nutrition and Metabolism, NOVA Medical School, Faculdade de Ciências Médicas, Universidade Nova de Lisboa, 1169-056 Lisboa, Portugal; 3 Royal Hospital for Children, Glasgow G51 4TF, UK; 4 Royal Victoria Infirmary, Newcastle upon Tyne NE1 4LP, UK; 5 Sheffield Children’s NHS Foundation Trust, Sheffield S10 2TH, UK; 6 Cardiff and Vale UHB, Cardiff CF14 4XW, UK; 7 University Hospitals Birmingham NHS Foundation Trust, Birmingham B15 2TH, UK; 8 Nottingham University Hospitals NHS Trust, Nottingham NG7 2UH, UK; 9 Cambridge University Hospitals NHS Foundation Trust, Cambridge CB2 0QQ, UK; 10 Royal Hospital for Children and Young People, Edinburgh EH16 4SA, UK; 11 University College London Hospitals NHS Foundation Trust, London WC1N 3BG, UK; 12 Guy’s and St Thomas’ NHS Foundation Trust, London SE1 7EU, UK; 13 North Bristol NHS Trust, Bristol BS10 5NB, UK; 14 University Hospitals of Leicester NHS Trust, Leicester LE1 5WW, UK; 15 Evelina London Children’s Healthcare, London SE1 7EH, UK; 16 Belfast Health and Social Care Trust, Belfast BT9 7AB, UK; 17 Great Ormond Street Hospital for Children NHS Foundation Trust, London WC1N 3JH, UK; 18 Sheffield Teaching Hospitals NHS Foundation Trust, Sheffield S5 7AU, UK; 19 Bradford Teaching Hospitals NHS Foundation Trust, Bradford BD5 0NA, UK; 20 Bristol Royal Hospital for Children, Bristol BS2 8BJ, UK; 21 CINTESIS, NOVA Medical School, Faculdade de Ciências Médicas, Universidade Nova de Lisboa, 1169-056 Lisboa, Portugal; 22 Reference Centre of Inherited Metabolic Diseases, Centro Hospitalar Universitario de Lisboa Central, 1169-045 Lisboa, Portugal; 23 University Hospitals Bristol NHS Foundation Trust, Bristol BS2 8HW, UK; 24 Salford Royal NHS Foundation Trust, Salford M6 8HD, UK

**Keywords:** Phenylketonuria, phenylalanine, protein, sapropterin, national consensus

## Abstract

Introduction: There is little practical guidance about suitable food choices for higher natural protein tolerances in patients with phenylketonuria (PKU). This is particularly important to consider with the introduction of adjunct pharmaceutical treatments that may improve protein tolerance. Aim: To develop a set of guidelines for the introduction of higher protein foods into the diets of patients with PKU who tolerate >10 g/day of protein. Methods: In January 2022, a 26-item food group questionnaire, listing a range of foods containing protein from 5 to >20 g/100 g, was sent to all British Inherited Metabolic Disease Group (BIMDG) dietitians *(n* = 80; 26 Inherited Metabolic Disease [IMD] centres). They were asked to consider within their IMD dietetic team when they would recommend introducing each of the 26 protein-containing food groups into a patient’s diet who tolerated >10 g to 60 g/day of protein. The patient protein tolerance for each food group that received the majority vote from IMD dietetic teams was chosen as its tolerance threshold for introduction. A virtual meeting was held using Delphi methodology in March 2022 to discuss and agree final consensus. Results: Responses were received from dietitians from 22/26 IMD centres (85%) (11 paediatric, 11 adult). For patients tolerating protein ≥15 g/day, the following foods were agreed for inclusion: gluten-free pastas, gluten-free flours, regular bread, cheese spreads, soft cheese, and lentils in brine; for protein tolerance ≥20 g/day: nuts, hard cheeses, regular flours, meat/fish, and plant-based alternative products (containing 5–10 g/100 g protein), regular pasta, seeds, eggs, dried legumes, and yeast extract spreads were added; for protein tolerance ≥30 g/day: meat/fish and plant-based alternative products (containing >10–20 g/100 g protein) were added; and for protein tolerance ≥40 g/day: meat/fish and plant-based alternatives (containing >20 g/100 g protein) were added. Conclusion: This UK consensus by IMD dietitians from 22 UK centres describes for the first time the suitability and allocation of higher protein foods according to individual patient protein tolerance. It provides valuable guidance for health professionals to enable them to standardize practice and give rational advice to patients.

## 1. Introduction

Phenylketonuria (PKU, OMIM 261600) is an autosomal recessive inborn error of amino acid metabolism. It is due to a deficiency or absence of the hepatic enzyme phenylalanine hydroxylase (PAH). More than 1200 pathological variants have been described [1], and most individuals with PKU have biallelic heterozygous variants in the gene encoding PAH [2]. PAH catalyses phenylalanine through an oxidative reaction with tetrahydrobiopterin (BH4), a co-factor, to form tyrosine. PAH deficiency leads to the accumulation of the amino acid phenylalanine (Phe) in the blood and brain [3]. If untreated, it causes permanent neurological disability associated with demyelination and changes in brain white matter, reduced neurotransmitter (e.g., adrenaline, epinephrine, and serotonin) synthesis, and impaired protein synthesis [4]. Classification is based on treatment needs. No intervention is required if untreated blood phenylalanine concentrations are <360 µmol/L; up to the age of 12 years, treatment is recommended if untreated blood phenylalanine is between 360–600 µmol/L, and lifelong treatment is recommended if untreated blood phenylalanine is >600 µmol/L at any age [3,5].

The traditional treatment is a phenylalanine-restricted diet. This excludes high protein/phenylalanine-containing foods such as meat, fish, eggs, milk and dairy products, and the artificial sweetener aspartame [6]. All patients require some dietary phenylalanine, and their individual phenylalanine tolerance is the amount of phenylalanine/natural protein that will maintain their blood phenylalanine within target therapeutic range. Most patients with a classical phenotype on dietary treatment tolerate less than 10 g/day natural protein, although people with milder PKU, who have some residual PAH activity, usually tolerate more [7].

In 2020, Evans et al. [8] reported the results of a dietetic consensus from UK IMD dietitians about the suitability of foods to include in a phenylalanine restricted diet when patients tolerate ≤10 g/day. It concluded that foods with a protein content of >0.5 g/100 g (exception: most fruits, some vegetables, and sauces made from fruit and vegetables) should be calculated/measured as part of a protein exchange system (the amount of food that provides 1 g protein/50 mg phenylalanine = 1 exchange). Recommended foods allocated as part of the protein/phenylalanine exchange system included breakfast cereals, potatoes, fruits and vegetables containing phenylalanine >75 mg/100 g, plant yoghurts, and milks. The Evans et al. [8] consensus did not consider patients with a protein tolerance of >10 g/day. The phenylalanine restricted diet is supplemented with a low phenylalanine/phenylalanine-free protein substitute, derived from either amino acids or glycomacropeptide, and they usually contain added vitamins, minerals, and long chain fatty acids. The dose of protein substitute is determined by the natural protein tolerance: when more natural protein is tolerated, a lower dose of protein substitute is required, thereby lowering the micronutrient intake [9]. Therefore, when natural protein tolerance is higher, it is important to ensure that a diverse range of nutrient-rich foods are eaten to meet micronutrient requirements.

Sapropterin dihydrochloride (sapropterin) is an adjunct pharmaceutical treatment that is effective in around 30% to 40% of people with PKU with residual enzyme activity [10]. It is a synthetic form of the naturally occurring cofactor tetrahydrobiopterin (BH4). High doses enhance the activity of the defective enzyme and thereby increase or restore the hydroxylation of phenylalanine [11]. In responsive patients, it is expected to lower blood phenylalanine concentrations [12,13,14] and improve phenylalanine tolerance [15,16]. Sapropterin responsiveness is identified by genetic testing and/or a sapropterin loading test [15]. Patients with two null mutations have complete enzyme deficiency and thus are not expected to respond to sapropterin. Patients with sapropterin-responsive or unclassified mutations are offered sapropterin responsive testing.

Along with sapropterin treatment, protein or phenylalanine intake is commonly increased by over 100% [17,18,19,20,21,22,23,24,25], with many patients tolerating around 20 to 40 g/day protein [26,27,28]. Much of the evidence has been generated from studies that originated from Spain, Italy, Turkey, and Germany, where PKU phenotype is generally milder [29]. Other studies report less dietary benefit [30,31,32,33,34,35]. Changes in protein and phenylalanine intake from the reported studies are given in Table 1.

Along with increasing protein tolerance, patients need guidance about the suitability of new and different food choices, including foods previously restricted or excluded. There are reports of nutrient imbalances in sapropterin-treated patients [9]. Thiele et al. reported that vitamin D, folic acid, iron, calcium, and iodine intakes did not meet requirements 2 years after sapropterin treatment [24]. Hennermann et al. [19] described low intakes of vitamin B12, calcium, and iron, but only in patients who had stopped their protein substitute. Brantley et al. reported a significant decrease in serum B12, dietary folate, and iron in paediatric patients after one year of sapropterin therapy [31]. In a systematic review, Rodrigues et al. [36] showed that in study cohorts that included sapropterin-treated patients, the overall body mass index (BMI) was significantly higher compared to controls. This did not occur in the diet-only treated cohort. This suggests that eating patterns on sapropterin may not be aligned with recommendations for health.

In PKU, there is little published practical advice about suitable food choices for patients with increasing protein tolerance. The UK has recently introduced sapropterin as an adjunct treatment for patients with PKU, and there are other potential pharmaceutical therapies emerging that may improve protein tolerance. To give consistent advice about the suitability of incorporating higher protein foods for patients with PKU who tolerate >10 g/day natural protein, UK specialist dietitians who were members of the British Inherited Metabolic Diseases Dietetic Group (BIMDG-DG) used Delphi methodology to agree upon a national consensus. This work was commissioned by the National Society for Phenylketonuria (NSPKU).

## 2. Materials and Methods

In January 2022, a non-validated questionnaire was developed (Supplementary Materials Table S1). This contained the 26 different higher-protein-containing food groups shown in Table 2. Using this questionnaire, dietitians were asked to select the minimum protein tolerance (either >10 g/day, ≥15 g/day, ≥20 g/day, ≥25 g/day, ≥30 g/day, ≥40 g/day, ≥50 g/day, or ≥60 g/day) at which they would consider introducing each of the 26 higher-protein-containing food groups. This questionnaire was sent to all BIMDG dietitians (*n* = 80; 26 adult/paediatric IMD centres) in the UK. All dietitians were trained specialist dietitians in IMD, and collectively, they had considerable experience in PKU care (mean 20 years; range: 2–43 years). The mean number of patients with PKU in each centre was 137 (range: 17–400), representing both adult and paediatric patients. Paediatric centres cared for smaller patient numbers, as transition age to adult care is at 16 years in the UK. The group had collectively published over 160 peer reviewed papers on PKU (>100 in the last 10 years).

Food groups were categorized by their protein content per 100 g: ≤10 g/100 g, 11–20 g/100 g, and >20 g/100 g (Table 2). There were 2 exceptions: dried lentils were in a group containing 11–25 g/100 g protein, and pasta, bread and flour in a group containing ≤12 g/100 g protein, as their protein content did not easily fit within other groups. Meat and fish products, diverse plant foods, and cheese (soft and hard) contained variable amounts of protein and were grouped into different categories according to their protein content (g/100 g of food). For example, soft cheese (<10 g/100 g protein) contains a lower concentration of protein than hard cheese (>20 g/100 g protein).

Protein-containing foods suitable for a protein tolerance of ≤10 g/day (e.g., milk, yoghurt/dairy desserts, hummus, coconut products, gluten-free breads) had been previously agreed and described as part of national BIMDG consensus statements [8] and are not discussed here. We did not consider the allocation of sweet foods such as regular chocolate, cakes, and biscuits. Our aim was to encourage healthier, nutrient-dense food choices for protein exchanges, as poor eating habits have been described when patients are allocated additional protein with adjunct therapies [9,24].

Prior to circulating the questionnaire to dietetic members of the BIMDG, the questionnaire was piloted by a group of 4 experienced dietitians who were part of the Medical Advisory Panel for the NSPKU (S.Ford, BC, MH, and AM). They checked the food categories and usability of the questionnaire. Some changes were made to the questionnaire structure and food categories in accordance with the results of the pilot testing.

BIMDG dietitians were given 7 weeks to complete the questionnaire and return it by email. A facilitator (SE) distributed the questionnaires, calculated responses, and reported results. When there was discrepancy in responses between dietitians from the same IMD centre, they were requested to discuss and reach consensus within their own team and submit one agreed response per team to the facilitator. The protein tolerance (g/day) that received the highest number of votes for inclusion of each of the 26 food groups was selected as the agreed majority. This became the minimum amount of protein a patient should tolerate before the food group could be introduced. Votes were also divided into paediatric and adult centres to assess differences between patient age groups.

A virtual meeting was held with the BIMDG dietitians in March 2022 to discuss the results of the initial questionnaire, to address any discrepancies, and agree on the protein tolerance levels for food group introduction, using Delphi methodology to reach consensus [37]. This methodology, a well-accepted qualitative communication technique, was used to gain a majority decision in a systematic way [37]. The meeting was chaired by a dietitian representing the NSPKU (AM).

## 3. Results

### 3.1. Voting Results

Responses were received from IMD dietitians representing 22 of 26 UK centres (85%). Eleven were paediatric and 11 adult centres. Four BIMDG centres (15%) did not respond; 2 centres had small patient numbers with PKU, and 2 centres were experiencing staff shortages so chose not to respond. In 3 centres, more than 1 dietitian replied. These centres were asked to reconsider and give a single combined response from their team of dietitians. Table 3 gives the results for each food group, showing the protein tolerance threshold that received the majority vote, which suggests the most suitable point to introduce the individual food group in a phenylalanine-restricted diet. None of the 26 food groups were considered suitable to introduce to patients with a protein tolerance of <15 g/day. The voting reflected a range of opinions on when to introduce the higher protein food groups; 9/26 food groups had a protein tolerance inclusion difference of 10–15 g across all respondents, 10/26 food groups had a difference of 20–25 g; and 7/26 food groups had a difference of 30–35 g. However, the median score and majority vote for the minimal protein tolerance (g/day) considered the most appropriate for inclusion was the same for 22 of 26 food groups. There were 4 exceptions: breads/bread products, feta cheese/cheese spreads, eggs, and nuts (protein content 10–20 g/100 g). Although the median score was for a higher minimum protein tolerance threshold than the majority vote percentage, all dietitians agreed to remain with the majority vote for the final decision.

Three food groups (nuts with a protein content of >20 g/100 g, flours with a protein content of approximately 20 g/100 g, and meat products with a protein content of 5–10 g/100 g) received a majority vote for inclusion when the patient protein tolerance threshold was 25 g/day or more. To simplify the guidance and number of protein tolerance categories, it was decided by all participants to advise that these be included in the diet when protein tolerance was 20 g/day or more.

### 3.2. Final Consensus Results

**Figure 1:**
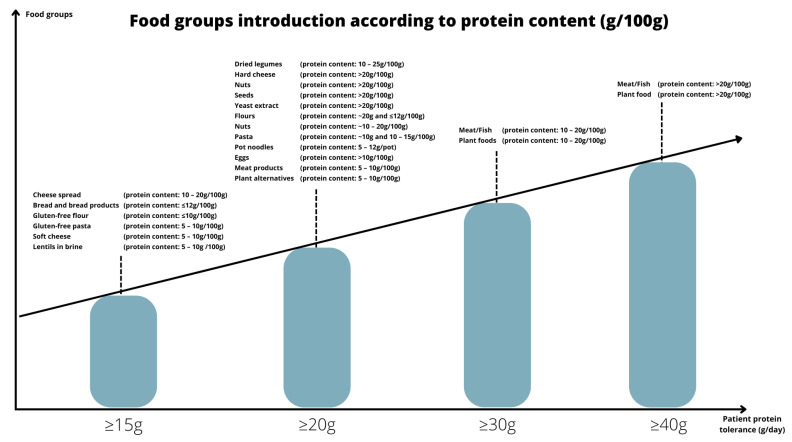
Food group introduction according to protein content (g/100g).

Table 4 and Figure 1 describe the final consensus reached by the BIMDG dietitians.

As no foods considered in the questionnaire were voted as appropriate for a protein tolerance below 15 g/day, the same restrictions on protein-containing foods will be recommended for patients on ≤15 g/day as for patients tolerating ≤10 g protein/day (Supplementary Materials Table S2).

### 3.3. Differences in Voting between Paediatric and Adult Care Centres

There were only minor differences between voting responses for paediatric versus adult care centres. Results are reported in Table 5. Paediatric centres voted to give higher-protein-containing food groups at a lower protein tolerance for 9/26 food groups; adult centres voted to give higher-protein-containing foods at a lower protein tolerance for 3/26 food groups; and for 14/26 food groups, there was no difference. The main discrepancies were observed for soft cheese, cheese spread, pasta containing approximately 10 g/100 g protein, dried legumes, hard cheese, seeds, and meat products containing between 5–10 g and 10–20 g/100 g protein, while most paediatric centres would introduce these at a lower protein tolerance. Conversely, for yeast extract, spreads, and nuts containing 10–20 g/100 g and >20 g/100 g protein, most adult centres would introduce these at a lower patient protein tolerance.

## 4. Discussion

This UK consensus by BIMDG dietitians, initiated by the NSPKU, identifies for the first time the suitability and inclusion of higher protein containing foods according to individual patient protein tolerance in PKU. It provides valuable guidance for health professionals to enable them to standardise practice and give rational advice to patients. The dietary benefits of sapropterin in responsive patients with PKU is variable, and usually, it does not facilitate the full liberalisation of natural protein intake. Food protein choices should be tailored around the individual patient, but priority should be given to the nutritional quality of the different foods. Balanced and nutritious food choices should be encouraged by health professionals, with the aim of achieving national guidance for healthy eating. When patients are first treated with sapropterin, they are suddenly faced with several new food options, even though they may only have limited knowledge about the nutritional content of foods that they have not eaten before. Without appropriate health professional advice, they may choose foods that are excessively high in protein, or of low nutritional quality, which will either impact their metabolic control or nutritional status.

The introduction of new and different food choices must consider the portion sizes that may be eaten in accordance with individual protein tolerance. The protein content of foods varies widely, from 0 g to 60 g/100 g of food [38]. Even with sapropterin, some patients may still only tolerate <20 g protein/day. This means that many high-protein foods such as eggs, meat, and cheese will still need severe restrictions or even avoidance, as a standard food portion size is unable to be tolerated [39]. For example, two medium eggs contain around 15 g protein; 60 g cheese, 12 g protein; 140 g fish, 28 g protein; and 90 g meat, around 18 g protein; so meat and fish portion sizes would be very small if given when protein tolerance is <20 g/day. However, foods such as bread, gluten-free pasta, cereals, soft cheese, milk, yoghurt, and canned lentils such as chickpeas can all be eaten in acceptable portion sizes and provide sources of good nutrition if protein tolerance does not exceed 20 g/day. When protein tolerance reaches 20 g/day it is reasonable to introduce eggs, hard cheese, and regular pasta; from 30 g/day to introduce meat, fish, and plant protein sources (containing 11–20 g/100 g protein); and from 40 g/day, meat and fish sources (containing >20 g/100 g protein). Many plant food protein sources such as Quorn, soya protein, or wheat/lentil meat alternatives are high in protein.

Little is known about how these consensus recommendations will affect the nutritional adequacy of the diet, considering that the nutrient profiles of different foods vary considerably. The BIMDG dietitians have recommended that desserts, cake, biscuits, popcorn, crisps, and other snacks be discouraged as part of regular protein sources and have not included these foods as part of this guidance, but there is a concern that patients will choose to eat these in large portion sizes if not actively discouraged. Food generates many emotions, and there is likely to be a patient/caregiver feeling that any food treats that have been in essence forbidden should now be given as part of the routine diet, as patients may have been deprived for many years. As it is recognised that poor eating behaviours established early in life often continue into adulthood, and so potentially increase the risk of obesity, type 2 diabetes, and cardiovascular disease [40], it is important to collect ongoing information about the nutritional quality of the diet and implement strategies to direct patients towards overall healthy and balanced nutrition.

Some patients may readily integrate new foods into their diets that will enhance their dietary quality. This was observed in patients who stopped dietary treatment in late childhood and were unable to successfully recommence protein restriction in adulthood [41]. However, many other patients will have reservations about eating higher protein foods. Adapting to a new protein tolerance is likely to take time, and rapidly increasing protein intake may negatively impact on the diet quality if it is not managed carefully. Eating behaviour expresses a range of variables i.e., food intake, choice, preference, hedonic response [liking], acceptance [intake], willingness to taste, neophobia, and historical dietary experiences [42]. Food preferences and neophobia are likely to be main drivers for food choice in children with PKU, but new social experiences, social modelling, and new food availability may affect this [43,44]. Long-term eating behaviours may be governed by the limited range of foods that were available early in life [42], and some patients may continue to eat the same familiar foods (but in larger portion sizes), irrespective of sapropterin treatment. As children mature and become increasingly independent of their parents for food choices, it is hoped that preferences will change. Willingness to alter eating behaviours is dependent on individual characteristics and type of dietary alterations, including choice of protein-containing foods [45]. It is probable that regular protein-containing foods such as bread, pasta, and cereals will taste and smell better than lower protein alternatives, so are likely to be readily accepted. The baseline level of protein tolerance may also affect acceptance of higher protein foods [46], i.e., patients who tolerate more protein when on dietary treatment only may eat a wider variety of protein-containing foods, but this requires further research.

Surprisingly, feeding behaviour with alternative therapies has not been addressed with rigorous scientific study in PKU. It is established that young children with PKU on dietary treatment only, display abnormal feeding patterns and show less interest in food, accompanied by food neophobia [47,48,49]. They may exhibit more food tantrums [50] and are even fearful of eating high-protein foods. Some children with PKU, usually with co-morbidities such as autism, demonstrate similar feeding behaviours to children with ‘avoidant/restrictive food intake disorder’ and provide additional difficulties [51]. There is also evidence of eating disorders, food neophobia, and adverse food attitudes in adults with PKU [7,52,53,54], but it is unknown how deep-seated feeding problems will affect lifelong eating patterns in PKU. Thiele showed in a small group of patients that after 2 years of sapropterin treatment, they still had a lower intake of milk, dairy, fish, and egg products compared to healthy controls, but they doubled their intake of potatoes, rice, and pasta [24].

Several studies report an association between nutrition literacy and its impact on healthy food choices [55,56,57,58]. Nutrition literacy encompasses food and nutrition knowledge, competence, and confidence in the ability to prepare food, and the skills required to understand and interpret complex information about foods and their nutrients [55]. In PKU, there is little information about patient or caregiver food literacy but ensuring that they have the necessary skills and knowledge to adjust their dietary intake with sapropterin therapy is essential. Adults with PKU with cognitive and executive function deficits present extra challenges, as some may struggle with many aspects of food literacy, particularly when their protein tolerance is modulated [41] and they step away from the comfort zone of their usual lifetime pattern of eating the same repetitive meals [54].

Thus, for both pre- and post- sapropterin introduction, an integrative framework addressing all aspects of patient/caregiver food literacy, together with clear guidance on the suitability of food choices according to protein tolerance, should be applied. The speed of introducing additional protein-containing foods should be conducted over months rather than weeks. This will allow time for patients to adapt to eating healthier food options and promote healthier relationships with food. Patient/caregiver food literacy, knowledge, and skills do not need to be at an expert level but should be enough to guide and support appropriate choices within their cultural norms. Education tools should be developed to aid food introduction on sapropterin, as well as interactive teaching sessions for patients/caregivers, aimed at improving food literacy not exclusively to the patients but also involving all the family. Overall, more controlled research on eating patterns in PKU, particularly upon alternative therapy introduction, is necessary. Longitudinal studies to assess how drug therapy changes the quality of the diet and impacts patients’ lives are essential.

This study has limitations. The Delphi methodology is a qualitative method of analysis, and it can be highly subjective. However, experienced UK IMD dietitians were involved in this process with consensus opinion being sought from each hospital dietetic team, which involved additional discussion and consideration. Although the total number of responses was small (*n* = 22 centres), this was representative of the majority of IMD centres across the UK. There was a wide range of patient protein tolerances suggested by dietitians for the appropriate introduction of higher-protein-containing foods. Some of this disparity may have reflected dietitians’ inexperience of caring for patients with PKU on higher protein tolerances, and the impact of the final decisions made should be monitored. This consensus did not consider reduction in protein substitute intake or reduction in the use of low-protein special foods with sapropterin. It is accepted that opinion may change with practice and clinical expertise and so it is recommended that this consensus be reviewed in 5 years.

## 5. Conclusions

There is little practical evidence on the suitability of inclusion of higher protein food choices at different patient protein tolerances in PKU. This UK BIMDG dietitians’ consensus document describes the introduction of higher protein foods according to individual protein tolerance and provides valuable guidance for health professionals to standardise practice and give consistent advice to patients throughout their lifetime. In PKU, initiatives to change eating behaviours should be conducted carefully, as pre-established eating habits may persist throughout life. Introduction of higher protein food choices should be tailored and individualised considering patient nutritional needs, preferences, and habits. This process should be conducted over time to allow healthy eating patterns to develop. Overall, an integrative approach, considering all aspects of nutritional literacy, should be adopted when changing and promoting healthy dietary practices in PKU. Both dietitians and the national patient associations have a vital role in supporting patients as they adjust to their new and changing treatments.

## Figures and Tables

**Table 1:** Changes in blood phenylalanine (Phe) levels and/or phenylalanine intake with sapropterin treatment.

Author, Year	Country	Number of Patients on Sapropterin Treatment (Age)	Length of Time of Follow-Up	Baseline Blood Phenylalanine (µmol/L)	Blood Phenylalanine (µmol/L) with Sapropterin	% Change in Blood Phenylalanine	Baseline Natural Protein/Phenylalanine Intake (mg/day)	Natural Protein/Phenylalanine Intake (mg/day) with Sapropterin	% Change in Natural Protein/Phenylalanine Intake
Lambruschini N et al. 2005 [20]	Spain	*n*= 11 (0.2–12.2 years)	1 year	382 ± 229	442 ± 141	+15.7% (n.s.)	356 ± 172	1546 ± 192	+334%
Burlina A and Blau N, 2009 [17]	Italy	*n*=12 (5.5 ± 4.7 years)	6 months–7 years	662 ± 221.4	N/A	N/A	498 ± 49	1475 ± 155	+196%
Singh RH et al., 2010 [22]	USA	*n* = 6 (8.7 ± 2.5 years)	24 months	N/A	N/A	N/A	421 ± 128	1470 ± 455	+249%
Hennermann JB et al., 2012 [19]	Germany	*n* = 18	48 ± 27 months	N/A	N/A	N/A	452 ± 201	1593 ± 647	+252%
Leuret O et al., 2012 [21]	France	*n* = 15 (39 ± 27 months)	23 (7–80) months	638 ± 176	240 ± 72	−62.4%	456 ± 181	1683 ± 627	+269%
Thiele AG et al., 2013 [25]	Germany	*n* = 8 (5 –16 years)	3 months	283 ± 145	304 ± 136	+7.4% (n.s.)	629 ± 476	2131 ± 1084	+239%
Thiele AG et al., 2015 [24]	Germany	*n* = 8 (10.5 ± 3.8 years)	2 years	262.2 ± 129.4	382.7 ± 148.1	+46% (s.s.)	493.2 ± 161.8	2021.9 ± 897.4	+310%
Longo N et al., 2015 [33]	USA	*n* = 504 (treatment group)	6 years	591 ± 382	392 ± 239	−33.7% (s.s.)	1000 ± 959	1197 ± 667	+20%
Aldamiz-Echevarria et al., 2013 [30]	Spain	*n* = 36 (5 ± 4.6 years)	2 years	255.2 ± 146.8	365.5 ± 226.5	+43.2%	29.9 (18.3–52.3) mg/kg/day	41.2 (22.9–48.9) mg/kg/day	+38%
		*n* = 10 (5.2 ± 3.1 years)	5 years	204.0 ± 143.9	289.6 ± 30.6	+42%	30.8 (24.6–54.8) mg/kg/day	38.1 (17.6–47.9) mg/kg/day	+24%
Tansek MZ et al., 2016 [23]	Slovenia	*n* = 9 (2–10 years)	2 years	200 [191–302]	190 [135–285]	−5% (n.s.)	620 [400–700]	2000 [1000–2000]	+223%
Feldmann R et al., 2017 [18]	Germany	*n* = 46 (24 paediatrics) Age: N/A	6 weeks	795.3 (340.8–1884)	N/A	N/A	13.8 (4.3–36.9) mg/kg/day	35.2 (11.9–81.5) mg/kg/day	+155%
Evers RAF et al., 2018 [32]	Netherlands	*n* = 21 (13.1 ± 9.2 years)	5 years	N/A	N/A	N/A	0.43 ± 0.28 g/kg/day	0.66 ± 0.26 g/kg/day	+54%
Brantley KD et al., 2018 [31]	USA	*n* = 18 (16.6 ± 10.3 years)	1 year	461.5 [366–539]	355 [231–427]	−23.1%	791 [529–2207]	1198 [993–1457]	+52%

Data is presented as: mean ± standard deviation, median (range) or median [interquartile range]; N/A = not applicable; n.s. = not significant; s.s. = statistically significant.

**Table 2:** Food group categories based on the protein content (g/100 g) of food (*n* = 26).

**Foods Containing Protein ≤10 g/100 g (*n* = 6 Food Groups)**
Canned pork sausages and baked beans, faggots, canned beef ravioli, sausage rolls (protein content 5–10 g/100 g) Plant-based meats/fish alternatives (containing vegetable/plant protein) Gluten-free pasta Soft cheese Lentils in water/brine/sauce (jars/canned) (e.g., baked beans, chickpeas, canned red, black, kidney, black-eyed, or mixed beans) Gluten-free flour, rye flour, rice flour, cornmeal/polenta
**Foods Containing Protein ≤12 g/100 g (*n* = 4 Food Groups)**
Bread (e.g., bread rolls, English muffins, bagels, ciabatta, wraps, croissants) Flour and starch (e.g., white, self-raising, wholemeal, chapatti, bread mixes, gram flour, spelt flour, sorghum flour, atta flour, cornmeal) Pot Noodles Pea pasta
**Foods Containing Protein between 11–15 g/100 g (*n* = 1 Food Group)**
Pasta (e.g., wheat, spelt)
**Foods Containing Protein between 11–20 g/100 g (*n* = 7 Food Groups)**
Chicken dippers, breaded chicken steaks, chicken nuggets, breaded chicken goujons, chicken fingers, chicken rolls, chicken burgers, chicken bites, pork pies, sausages, chopped pork, meat burgers (protein content 11–20 g/100 g) Breaded cod fillets, fish fingers, fishcakes, tinned fish in sauce, fish paste (protein content 11–20 g/100 g) Plant-based meats/fish alternatives (containing soya, lentils, pea protein, tofu, Quorn-based products) (protein content 11–20 g/100 g) Hens eggs (boiled, poached, fried, scrambled, omelette) Cheese spread, feta, cottage cheese Nuts (e.g., pine, Brazil, cashew, pecan, walnuts) Flours (e.g., chickpea, almond, coconut, pease meal, chestnut flour)
**Foods Containing Protein between 11–25 g/100 g (*n* = 1 Food Group)**
Dried legumes/pulses, e.g., lentils, chickpeas, dried peas, split peas, beans (baked, red, black)
**Foods Containing Protein >20 g/100 g (*n* = 7 Food Groups)**
Meat (lamb, beef, pork, ham, bacon, chicken, turkey, duck, game, beef jerky, corned beef, beef or higher protein meat burgers, meat paste, meat pies, burgers, offal) (protein content >20 g/100 g) Fish (all varieties including shellfish, frozen) (protein content >20 g/100 g) Plant-based meat/fish alternatives (containing soya, lentils, pea protein, Quorn-based products) (protein content >20 g/100 g) Hard cheese Nuts (e.g., peanuts, peanut butter, almonds, pistachio) Seeds (e.g., sesame, pumpkin, chia, poppy, flax) Yeast extract spreads (e.g., Marmite, Vegemite, Bovril)

**Table 3:** Results outlining the majority votes from **the BIMDG Dietetic Teams** for the recommended patient protein tolerance threshold (g/day) for the introduction of different food groups.

Food Group (Protein Content) (g/100 g)	Protein Tolerance (g/day) Considered the Most Suitable Point to Introduce the Protein-Containing Food Group based on majority votes	% (*n*) of Centres Voting for the Protein Tolerance Level	The Overall Range of Protein Tolerance (g/day)	Overall Median Protein Tolerance (g/day)
Gluten-free pasta (5–10 g)	≥15 g	55 (12/22)	10–20	15
Lentils in water/brine (5–10 g)	≥15 g	62 (13/21)	10–20	15
Soft cheese (5–10 g)	≥15 g	45 (10/22)	10–30	15
Gluten-free flour (<10 g)	≥15 g	55 (12/22)	10–25	15
Bread and bread products (≤12 g)	≥15 g	45 (10/22)	15–30	20
Feta cheese/cheese spread (10–20 g)	≥15 g	41 (9/22)	10–40	20
Plant meat/fish alternatives (5–10 g)	≥20 g	62 (13/21)	10–30	20
* Pasta (~10 g)	≥20 g	43 (9/21)	15–30	20
* Flours (≤12 g)	≥20 g	41 (9/22)	10–30	20
Pot Noodles (5–12 g/pot)	≥20 g	65 (13/20)	15–25	20
* Pasta (11–15 g)	≥20 g	45 (10/22)	15–30	20
Eggs (10–20 g)	≥20 g	36 (8/22)	15–40	25
Nuts (10–20 g)	≥20 g	45 (10/22)	15–30	23
Dried legumes (10–25 g)	≥20 g	33 (7/21)	15–30	20
Hard cheese (>20 g)	≥20 g	41 (9/22)	15–50	20
Seeds (>20 g)	≥20 g	33 (7/21)	10–40	20
Yeast extract spreads (>20 g)	≥20 g	40 (8/20)	10–40	20
Meat products (5–10 g)	≥25 g	35 (7/20)	15–40	25
* Flours (~20 g)	≥25 g	41 (9/22)	10–40	25
Nuts (>20 g)	≥25 g	45 (10/22)	15–50	25
Meat (10–20 g)	≥30 g	52 (11/21)	20–40	30
Fish (10–20 g)	≥30 g	52 (11/21)	20–40	30
Plant meat/fish alternatives (10–20 g)	≥30 g	71 (15/21)	20–40	30
Meat (>20 g)	≥40 g	62 (13/21)	30–50	40
Fish (>20 g)	≥40 g	52 (11/21)	25–50	40
Plant meat/fish alternatives (>20 g)	≥40 g	48 (10/21)	20–50	40

Note: not every centre provided a response for every food. * Flours and pasta included 2 food groups each due to differing protein ranges associated with cereal origin e.g., flours containing protein ~20 g/100 g included chickpea and almond flour, and flours containing protein ≤12 g/100 g included wheat and spelt flours; pasta containing protein ~10 g/100 g included pea pasta, and pasta containing protein 11–15 g/100 g included wheat and spelt pasta.

**Table 4:** UK BIMDG dietitian consensus for the allocation of food groups according to individual patient protein tolerance.

Patient Daily Protein Tolerance	Foods/Food Groups Allocated	Protein Content/100 g
≥15 g protein/day	Lentils in brine	5–10 g
	Soft cheese	5–10 g
	Cheese spread	10–20 g
	Bread and bread products	≤12 g
	Gluten-free flour	≤10 g
	Gluten-free pasta	5–10 g
≥20 g protein/day	Nuts	>20 g
	Flours *	~20 g and ≤12 g
	Meat products	5–10 g
	Plant alternatives	5–10 g
	Nuts	~10–20 g
	Hard cheese	>20 g
	Pot Noodles	5–12 g/pot
	Pasta *	~10 g and 10–15 g
	Seeds	>20 g
	Yeast extract	>20 g
	Eggs	10–20 g
	Dried legumes	10–25 g
≥30 g protein/day	Meat/Fish	>10–20 g
	Plant foods	>10–20 g
≥40 g protein/day	Meat/Fish	>20 g
	Plant foods	>20 g

* Flours and pasta included 2 food groups each due to differing protein ranges associated with cereal origin e.g., flours containing protein ~20 g/100 g included chickpea and almond flour, and flours containing protein ≤12 g/100 g included wheat and spelt flours; pasta containing protein ~10 g/100 g included pea pasta, and pasta containing protein 11–15 g/100 g included wheat and spelt pasta.

**Table 5:** The highest number of votes given by paediatric and adult centres for inclusion of each of the food groups according to patient protein tolerance.

Food Group (Protein Content) (g/100 g)	Protein Tolerance Level with the Highest Number of Votes	% (*n*) of Centres Voting for the Tolerance Level		Protein Tolerance Range		Median Minimal Protein Tolerance (g/day)	
		Paediatric centre	Adult centre	Paediatric centre	Adult centre	Paediatric centre	Adult centre
Gluten-free pasta (5–10 g)	15 g	45 (5)	64 (7)	10–20	10–20	15	15
Lentils in water/brine (5–10 g)	15 g	70 (7)	54 (6)	10–20	10–20	15	15
Soft cheese (5–10 g)	15 g	54 (6)	36 (4)	10–20	10–30	15	17.5
Gluten-free flour (<10 g)	15 g	64 (7)	45 (5)	10–25	10–20	15	15
Bread and bread products (≤12 g)	15 g	45 (5)	45 (5)	15–25	15–30	20	20
Cheese spread/feta (10–20 g)	15 g	63 (7)	18 (2)	10–40	20–30	15	20
Plant meat/fish alternatives (5–10 g)	20 g	60 (6)	64 (7)	10–30	15–30	20	20
Pasta (~10 g)	20 g	27 (3)	60 (6)	15–25	20–30	15	20
Flours (≤12 g)	20 g	36 (4)	45 (5)	10–30	15–30	20	20
Pot Noodles (5–12 g/pot)	20 g	80 (8)	50 (5)	15–25	15–25	20	20
Pasta (10–15 g)	20 g	45 (5)	45 (5)	15–30	15–30	20	20
Eggs (10–20 g)	20 g	36 (4)	36 (4)	15–40	15–40	25	25
Nuts (10–20 g)	20 g	36 (4)	55 (6)	20–30	15–30	25	20
Dried legumes (10–25 g)	20 g	30 (3)	36 (4)	15–25	15–30	15	20
Hard cheese (>20 g)	20 g	55 (6)	27 (3)	15–40	15–50	20	25
Seeds (>20 g)	20 g	30 (3)	36 (4)	15–40	10–40	17.5	20
Yeast extract spreads (>20 g)	20 g	60 (6)	20 (2)	10–40	10–40	20	17.5
Meat products (5–10 g)	25 g	40 (4)	30 (3)	15–40	15–40	25	30
Flours (~20 g)	25 g	27 (3)	55 (6)	10–40	15–40	25	25
Nuts (>20 g)	25 g	64 (7)	27 (3)	20–50	15–50	25	20
Meat (10–20 g)	30 g	60 (6)	45 (5)	20–40	30–40	30	40
Fish (10–20 g)	30 g	50 (5)	55 (6)	20–40	20–40	30	30
Plant meat/fish alternatives (10–20 g)	30 g	70 (7)	72 (8)	20–40	20–40	30	30
Meat (>20 g)	40 g	50 (5)	72 (8)	30–50	40–50	40	40
Fish (>20 g)	40 g	20 (2)	82 (9)	25–50	40–50	40	40
Plant meat/fish alternatives (>20 g)	40 g	40 (4)	55 (6)	20–50	20–50	35	40

## Data Availability

Not applicable.

## Supplementary Materials

The following supporting information can be downloaded at https://www.mdpi.com/article/10.3390/nu14234987/s1, Table S1: Questionnaire—Protein Cut Off Points; Table S2: Final consensus for the allocation of food groups according to individual patient protein tolerance (including ≥10 g/day).
