# Supplementary material for: Suitability and Allocation of Protein-Containing Foods According to Protein Tolerance in PKU: A 2022 UK National Consensus
Source: Nutrients. 2022 Nov 24;14(23):4987. doi: 10.3390/nu14234987 (PMC9736047; doi:10.3390/nu14234987)
Supplement: Supplementary file 1 [file nutrients-14-04987-s001.zip › nutrients-1976192-supplementary.pdf]

SUPPLEMENTARY DATA – Table S1

Protein cut off points

Some patients with PKU (with or without sapropterin treatment) tolerate over 10g/protein daily. The types of food we recommended in their diet will vary according to individual protein tolerance. At what patient daily protein tolerance should the following foods be included in the diet? This is particularly important to determine now that we can use sapropterin with patients.

What is your opinion? Please choose a protein cut off point for each food category according to the daily protein tolerance. Put a **x** in the box that you would consider the protein tolerance cut off point when you consider it reasonable (e.g., a useful food portion could be eaten) to allow any of the following foods

FOOD GROUPS	Allowed 10g protein/ day	Allowed 15g protein/ day	Allowed 20g protein/ day	Allowed 25g protein/ day	Allowed 30g protein/ day	Allowed 40g protein/ day	Allowed 50g protein/ day	Allowed 60g protein/ day
Meat (lamb, beef, pork, ham, bacon, chicken. turkey, duck, game, beef jerky, corned beef, beef or higher protein meat burgers, meat paste, meat pies). Offal (liver, kidney, tongue). Burgers<br>**Definition contains protein over 20g/100g -**								
Chicken dippers, breaded chicken steaks, chicken nuggets, breaded chicken goujons, chicken fingers, chicken roll, chicken burgers, chicken bites, pork pies,								

sausages, chopped pork. Billie bear meat, small meat protein burgers<br><br>**Definition contains protein 10-20g/100g**								
Canned Pork sausages and baked beans, faggots, canned beef ravioli, sausage rolls<br><br>**Definition contains protein 5--10g/100g**								
Fish (all varieties including shellfish, frozen)<br><br>**Definition contains protein over 20g/100g**								
Breaded cod fillets, fish fingers, fishcakes, tinned fish in sauce, fish paste<br><br>**Definition contains protein 10-20g/100g**								
**Plant meats/fish**<br><br>Containing soya, lentils, pea protein, Quorn based products<br><br>**Definition contains protein over 20g/100g**								
**Plant meats/fish alternatives**<br><br>Containing soya, lentils, pea protein, tofu, Quorn based products<br><br>**Definition contains protein over 10-20g/100g**								
**Plant meats/fish**<br><br>Containing vegetable/plant protein<br><br>**Definition contains protein 5--10g/100g**								

Hens Eggs – boiled, poached, fried, scrambled, omelette<br><br>**Definition contains protein over 10-20g/100g**								
Hard Cheese<br><br>**Definition contains protein over 20g/100g**								
Soft cheese<br><br>**Definition contains protein 5--10g/100g**								
Cheese spread, feta, cottage cheese<br><br>**Definition contains protein over 10-20g/100g**								
Nuts e.g. peanuts, peanut butter, almonds, pistachio<br><br>**Definition contains protein over 20g/100g**								
Nuts e.g. pine, brazil, cashew, pecan, walnuts<br><br>**Definition contains protein over 10-20g/100g**								
Seeds e.g. sesame, pumpkin, chia, poppy, flax<br><br>**Definition contains protein over 20g/100g**								
Yeast extract spreads e.g. Marmite, Vegemite, Bovril<br><br>**Definition contains protein over 20g/100g**								

Bread e.g., bread roll, English muffin, bagels, ciabatta, wrap, croissants<br>**Definition contains protein ≤ 12g/100g**								
Flours e.g., chickpea, almond, coconut, peasemeal, chestnut flour<br>**Definition contains protein around 20g/100g**								
Flour and starch e.g., white, self-raising, wholemeal, chapatti, bread mixes, gram flour, spelt flour, sorghum flour, atta flour, spelt flour, cornmeal<br>**Definition contains protein ≤ 12g/100g**								
Gluten free flour, rye flour, rice flour, cornmeal/polenta<br>**Definition contains protein < 10g/100g**								
Pasta e.g., pea<br>**Definition contains protein 10g/100g**								
Pasta.e.g. wheat, spelt<br>**Definition contains protein 10-15g/100g**								
Gluten free pasta<br>**Definition contains protein 5--10g/100g**								
Pot Noodles<br>**Definition contains protein 5--12g per pot**<br>NOT high protein pots								
Dried Legumes/pulses e.g., Lentils, chickpeas, dried peas, split peas. Beans – baked, red & black,								

**Definition contains protein 10-25g/100g**<br><br>This is not the same as lentils canned in water								
Lentils in water/brine/sauce (jars/canned)<br><br>Baked beans<br><br>Chickpeas, tinned red, black, kidney beans, mixed beans, black-eyed beans in water/brine/sauce<br><br>**Definition contains protein 5-10g/100g**								

SUPPLEMENTARY DATA – Table S2

Final consensus for the allocation of food groups according to individual patient protein tolerance (including ≥10g protein/day)

Patient daily protein tolerance	Foods/Food Groups allocated	Protein content/100g
≥10g protein/day	Milk (animal)	<5g/100ml
	Yoghurt (animal)/dairy desserts	<10g
	Hummus	5-10g
	Coconut products	<10g
	Gluten-free breads	≤5g
≥15g protein/day	Lentils in brine	5-10g
	Soft cheese	5-10g
	Cheese spread	10-20g
	Bread and bread products	≤12g
	Gluten-free flour	≤10g
	Gluten-free pasta	5-10g
≥20g protein/day	Nuts	>20g
	Flours	~20g and ≤12g
	Meat products	5-10g
	Plant alternatives	5-10g
	Nuts	~10-20 g
	Hard cheese	>20g
	Pot noodles	5-12g/pot
	Pasta	~10g and 10-15g
	Seeds	>20g
	Yeast extract	>20g
	Eggs	10-20g
	Dried legumes	10-25g
≥30g protein/day	Meat/Fish	>10-20g
	Plant foods	>10-20g
≥40g protein/day	Meat/Fish	>20g
	Plant foods	>20g
